# A novel mutation of the *ACADM *gene (c.145C>G) associated with the common c.985A>G mutation on the other *ACADM *allele causes mild MCAD deficiency: a case report

**DOI:** 10.1186/1750-1172-5-26

**Published:** 2010-10-05

**Authors:** Anne-Frédérique Dessein, Monique Fontaine, Brage S Andresen, Niels Gregersen, Michèle Brivet, Daniel Rabier, Silvia Napuri-Gouel, Dries Dobbelaere, Karine Mention-Mulliez, Annie Martin-Ponthieu, Gilbert Briand, David S Millington, Christine Vianey-Saban, Ronald JA Wanders, Joseph Vamecq

**Affiliations:** 1 Department of Biochemistry and Molecular Biology, Laboratory of Hormonology, Metabolism-Nutrition & Oncology (HMNO), Center of Biology and Pathology (CBP) Pierre-Marie Degand, CHRU Lille, 59037 Lille, France; 2 Research Unit for Molecular Medicine, Institute of Clinical Medicine, Aarhus University, 8200 Aarhus N, Denmark; 3 Department of Biochemistry and Molecular Biology, University of Southern Denmark, 5230 Odense M, Denmark; 4 Biochemistry Laboratory, CHU Bicêtre, 94275 Le Kremlin-Bicêtre, France; 5 Metabolic Biochemistry Unit, Hôpital Necker-Enfants Malades, Paris, France; 6 Pediatric Neurology Epilepsy Branch, CHU Rennes, 35203 Rennes, France; 7 Medical Reference Center for Inherited Metabolic Diseases, Jeanne de Flandres Hospital, CHRU Lille, 59037 Lille, France; 8 Mass Spectrometry Application Laboratory, University of Lille 2, 59045 Lille, France; 9 Department of Paediatrics, Duke University Medical Center, Durham, NC 27713, USA; 10 Center of Biology and Pathology East, CHU Lyon, 69677 Bron, France; 11 Laboratory of Genetic Metabolic Diseases, Academic Medical Center at the University of Amsterdam, Meibergdreef 9, 1105 AZ Amsterdam, The Netherlands; 12 Inserm, External Laboratory, Department of Prof. Nicole Porchet, HMNO, Center of Biology and Pathology (CBP) Pierre-Marie Degand, CHRU Lille, 59037 Lille, France

## Abstract

A female patient, with normal familial history, developed at the age of 30 months an episode of diarrhoea, vomiting and lethargy which resolved spontaneously. At the age of 3 years, the patient re-iterated vomiting, was sub-febrile and hypoglycemic, fell into coma, developed seizures and sequels involving right hemi-body. Urinary excretion of hexanoylglycine and suberylglycine was low during this metabolic decompensation. A study of pre- and post-prandial blood glucose and ketones over a period of 24 hours showed a normal glycaemic cycle but a failure to form ketones after 12 hours fasting, suggesting a mitochondrial β-oxidation defect. Total blood carnitine was lowered with unesterified carnitine being half of the lowest control value. A diagnosis of mild MCAD deficiency (MCADD) was based on rates of 1-^14^C-octanoate and 9, 10-^3^H-myristate oxidation and of octanoyl-CoA dehydrogenase being reduced to 25% of control values. Other mitochondrial fatty acid oxidation proteins were functionally normal. *De novo *acylcarnitine synthesis in whole blood samples incubated with deuterated palmitate was also typical of MCADD. Genetic studies showed that the patient was compound heterozygous with a sequence variation in both of the two *ACADM *alleles; one had the common c.985A>G mutation and the other had a novel c.145C>G mutation. This is the first report for the *ACADM *gene c.145C>G mutation: it is located in exon 3 and causes a replacement of glutamine to glutamate at position 24 of the mature protein (Q24E). Associated with heterozygosity for c.985A>G mutation, this mutation is responsible for a mild MCADD phenotype along with a clinical story corroborating the emerging literature view that patients with genotypes representing mild MCADD (high residual enzyme activity and low urinary levels of glycine conjugates), similar to some of the mild MCADDs detected by MS/MS newborn screening, may be at risk for disease presentation.

## Introduction

Mitochondrial fatty acid oxidation defects (FAOD) are rare inherited diseases with clinical presentation ranging from severe outcome to asymptomatic status. Diagnosis of FAOD requires specific investigations including biochemical and genetic studies. The most common FAOD is represented by a deficiency in the activity of the medium-chain acyl-CoA dehydrogenase (MCAD, EC 3.1.2.20) protein, which is coded by the *ACADM *gene (chromosome 1p31) [1-4]. First reported in 1976 [5] and biochemically characterized in 1982 [6], MCAD deficiency (MCADD) has since been extensively described. Point mutations in the *ACADM *gene were reported early [7,8] and many mutations have been documented subsequently, in part as a result of the large development of screening strategies [9-18]. As a general rule, these mutations lead to either truncated/absent protein synthesis, or replacement of one amino acid by another with a consequent drop in the activity of the mutant protein. The most common *ACADM *mutation is c.985A>G, which causes the replacement of a lysine by a glutamate at position 329 of the precursor protein (position 304 of the mature protein) [3,17], being for this reason also referred to as the K329E (or K304E) protein variant. Eighty percent of symptomatic MCADD patients of European origin are homozygous for this mutation, and a further 18% of symptomatic MCADD are associated with the c.985A>G mutation in one allele and a rare mutation in the other allele [1,3,19,20]. Mutations other than c.985A>G may predominate in non-European populations [21,22].

MCADD is inherited as an autosomal recessive trait [2,3,23,24]. Like other genetic β-oxidation defects, MCADD may cause in early childhood life-threatening events like hypoketotic/hypoglycaemic comas occurring during the course of catabolic states which disrupt the body energetic production/expense balance (infectious episodes, for instance) [2,3,23,24]. In fact, MCADD may present phenotypically as either a classical (severe) or a mild form. In mild MCADD, the residual MCAD activity is intermediate between that observed in classical MCADD (usually less than a few percent) and their heterozygous carriers (around 50%) [25], the classical form of the disease resulting mainly from homozygosity for the c.985A>G mutation as mentioned above. The residual activity of the *ACADM *gene product and hence its impact on cell metabolism and patient clinical presentation depends on a series of factors including protein misfolding, MCAD tetramer stability, and thermosensitivity of the mutant protein [3,26-28] along with contribution from the overlapping activities of other mitochondrial acyl-CoA dehydrogenases and efficacy of L-carnitine-based detoxification. In turn, a toxic "gain-of-function" pathogenesis has been also proposed for some MCAD variants [3].

Febrile episodes may negatively impact the disease outcome, especially when the mutant protein is thermosensitive, which is the case for the most common K304E protein variant [28]. The patient has then to face concomitantly not only the increased energy demand but also the sudden lowering of the fatty acid oxidation-derived energy production secondary to a drop in residual MCAD activity. This might explain why one quarter of the patients with classical MCADD do not survive their first life-threatening episode and why this disease is present in the etiology list for sudden infant death syndrome [29-31].

Like patients with classical MCADD, patients with mild MCADD may develop metabolic decompensation in their early childhood. They may have to cope throughout life with neurological sequels or other irreversible lesions caused by early childhood metabolic decompensation. These sequels do not necessarily reflect the MCADD genotype but rather are the consequences of the life threatening episode before medical intervention. Recent work, however, suggests that the link between susceptibility to develop metabolic decompensation and MCADD patient genotype is not straightforward and that this susceptibility is largely dependent on environmental triggers such as starvation stress, infection and/or fever [3,18]. We report here on a patient with mild MCADD having undergone a severe metabolic decompensation and subsequent clinical sequels. Genetic studies revealed a previously unreported mutation (c.145C<G) in one of the *ACADM *alleles combined with the common c.985A>G mutation in the other allele. Biochemical investigations of the patient are reported along with clinical data from the same time and an assessment of the impact of the new mutant protein variant is discussed.

## Materials and methods

### Solvents, reagents and internal calibrators for *in vitro* biological explorations

Chemical reagents and solvents were HPLC grade and were obtained from Merck, acetonitrile from JT Baker, 1-butanol and methanol from Prolabo, and stable isotopes including [16-^2^H_3_, 15-^2^H_2_]-palmitate (substrate), [16-^2^H_3_]-pamitoylcarnitine, [8-^2^H_3_]-octanoylcarnitine and [3-^2^H_3_]-propionylcarnitine (internal standards) from CDN-Isotopes Inc. L-carnitine and other chemicals were from Sigma (St Louis, MO).

### Determinations of body fluid organic acids, acylglycines, carnitine (total, unesterified and esterified forms) and acylcarnitines

Trimethylsilyl derivatives of organic acids were quantified using capillary gas chromatography (GC) coupled to mass spectrometry (MS), and total and unesterified carnitine levels were determined as previously described [32,33]. Individual acylcarnitines in body fluids were analyzed by fast atom bombardment (FAB)-MS or MS-MS of a blood spot on filter paper (Guthrie card) [33-35].

### Preparation of lymphocytes and culture of fibroblasts

Mononuclear cells were isolated from 10 mL of blood, using a Ficoll-Paque procedure. The pellet was suspended in Dulbecco's phosphate buffered saline with 0.9 mM Ca^2+ ^and 0.5 mM Mg^2+^, pH 7.35 (PBS), in order to adjust total cell proteins of the cell suspension to 1 ± 0.4 mg/ml for enzymatic determinations.

Skin fibroblasts were cultured as previously described [36,37].

### Fatty acid oxidation rate studies on lymphocytes and fibroblasts

Tritiated water release experiments [38] were performed in triplicate, in 24-well microtiter plates. [9, 10(n)-^3^H] palmitic and myristic acids bound to albumin (0.5 mg/ml) were used as substrates, at a final concentration of 100 μmol/l in PBS.

The capacity to oxidize ^14^C lapelled substrates was examined essentially as described previously [36,37].

### Measurement of individual enzymes of fatty acid oxidation

Mitochondrial acyl-CoA dehydrogenases were assayed by the ETF reduction assay [38]. Other fatty acid oxidation proteins were assayed by well standardized procedures [39,40].

### Genetic studies

Preparation of DNA samples of blood origin and PCR, purification and sequence analysis of exons of the *ACADM *gene were performed as described previously [12].

### Procedure relative to *de novo* synthesis of acylcarnitines by whole blood samples

*De novo *synthesis of acylcarnitines by whole blood samples incubated with deuterated palmitate was monitored essentially as described in Dessein et al. [34] with the ESI tandem mass spectrometry performed with either a Perkin Elmer mass or a Micromass Quattro PremierTM mass spectrometer.

## Case description

### First clinical symptoms and biological data

The patient was born normally as a result of a twin pregnancy, her twin brother being clinically healthy. Her eldest brother has also been healthy. She had a normal gestation and familial history.

At the age of 30 months, she was hospitalized for diarrhea without fever, vomiting and lethargy. Electroencephalogram and brain scanner examinations were normal. Glycaemia was normal as well as aminoacidogram. Discrete biological liver cytolysis was noticed with GOT and GPT increased at 45 and 90 IU/l, respectively (upper control values at 30 IU/l) along with echographical signs of mild hepatomegaly. Viral and bacterial serology was negative. This episode spontaneously resolved in a couple of days, and the patient recovered rapidly to a healthy status and normal life activities.

At the age of 3 years, she re-iterated an episode of vomiting which was sub-febrile (38.5°C) and followed by a coma, requiring a new hospitalization. On this occasion, marked hypoglycaemia (0.17 g/L) was documented and the patient presented with tonic-clonic seizures on the right side which persisted for 3 days and was treated by combined phenobarbital and phenytoin. The recovery of this *status epilepticus *was progressive. The child developed, however, sequels including a deficit of the right hemi-body, delay in the language acquisition, and electroencephalographic abnormalities in the rear area of the left hemisphere. The biological analyses revealed a liver cytolysis (increased blood levels of GPT [96 IU/l] and GOT [134 IU/l]) without signs of hepatic failure. A study of pre- and post-prandial blood glucose and ketones over a period of 24 hours showed normal glycaemias but a failure to form ketones after 12 hours fasting. This prompted a study of mitochondrial fatty acid oxidation *via *metabolite analyses in body fluids samples and biochemical measurements in cultured skin fibroblasts and lymphocytes. The diagnostic biological/biochemical investigations were completed by genetic studies and functional assessment of *de novo *acylcarnitine synthesis by whole blood samples.

### Diagnostic biological and biochemical explorations

#### - Metabolite analyses

The results of metabolite analyses are given in Table 1. Urinary organic acid profiles obtained during the comatose metabolic crisis showed increased medium-chain dicarboxylic acids but neither hexanoylglycine nor suberylglycine were detected by routine GC-MS analysis. 3-Hydroxybutyrate was increased in the urine indicating some ability to form ketone bodies. The patient presented with hypocarnitinemia affecting both total and unesterified carnitine levels which were reduced to half of the control values. This hypocarnitinemia was treated by oral L-carnitine supplementation (100 mg/kg/day) maintained throughout life (with a dose limit of 1.5 g/day). Several other metabolite analyses in blood and urine were performed when the patient was under L-carnitine therapy. Under these conditions, blood total and unesterified carnitine levels were normalized, and the esterified to unesterified carnitine ratio was at the upper control limit. Blood acylcarnitine analysis showed an increase in octanoylcarnitine with a C8/C10 acylcarnitine ratio higher than 5 and evocative of a defect in octanoate oxidation. In the patient urine, C8 acylcarnitines represented more than half of the total acylcarnitine content in contrast to the normal urine samples in which, even under carnitine load, the major acylcarnitine is acetylcarnitine [33].

**Table 1:** Major metabolite analysis data in patient

Type of analysis	Metabolites	Patient	Controls
		(μmol/mmol creatinine)	(μmol/mmol creatinine)
URINARY ORGANIC ACIDS UPON METABOLIC CRISIS	3-hydroxybutyrate	930	< 56
	Dicarboxylic acids		
	Adipic acid	894	< 21
	Suberic acid	254	< 8
	Sebacic acid	138	< 2
	Hexanoylglycine	< 2	< 2
	Suberylglycine	< 2	< 2
		(μmol/l)	(μmol/l)
CARNITINE FRACTIONS IN BLOOD	Total carnitine	22 (82**^c ^**)	34.6-83.6
	Unesterified carnitine	12 (45**^c ^**)	24.3-83.6
	Esterified carnitine	10 (37**^c ^**)	4.0-28.3
	Esterified/unesterified carnitine ratio	0.83 (0.82**^c^**)	0.11-0.83
		(μmol/mmol creatinine)	(μmol/mmol creatinine)
BLOOD ACYLCARNITINES (DRIED BLOOD SPOT)**^c^**	Octanoyl-carnitine	0.90**^a^**	< 0.3 (n = 42)
	C8/C10 acylcarnitine ratio	> 5.1**^b^**	
URINARY ACYLCARNITINES**^c^**			
GS/MS analysis of lactones (chemical ionization)	Octanoyl-carnitine	63%	Acetylcarnitine as the major acylcarnitine
	Hexanoyl-carnitine	24%	
	Acetyl, isobutyryl, butyryl, isovaleryl and 2-methyl butyryl- carnitine	Traces	
Direct analysis of acylcarnitines by FAB/MS	Octanoyl-carnitine	55%	Acetylcarnitine as the major acylcarnitine
	Hexanoyl-carnitine	10%	
	Nonanoylcarnitine	5%	
	Acetyl, isobutyryl, butyryl, isovaleryl, decanoyl and 2-methylbutyrylcarnitine	Traces	

Range values for c.985A>G homozygotes are **^a ^** 0.5-15.6 (n = 53) and **^b ^** 5.6-87 (n = 62).

**^C ^** Analytic determinations performed in patient under L-carnitine therapy (100 mg/kg/day).

#### - Biochemical determinations

Fatty acid oxidation rates measured on intact lymphocytes (Table 2) were within control range for butyrate and palmitate labeled at carbon 1, whereas octanoate oxidation was deficient. Using either myristate or palmitate labeled by tritium substitution of carbons 9 and 10 of the fatty acid indicated residual fatty acid oxidation activities of 11 and 24% of controls, respectively.

**Table 2:** Measurement of fatty acid oxidation rates in lymphocytes.

Metabolic steps	Substrates	Patient	Control tested in the same run	Controls(n = 40)
		(nmol/hr.mg protein)	(nmol/hr.mg protein)	(nmol/hr.mg protein)
Mitochondrial oxidations assayed				
- by carbon dioxide production	[1-^14^C]-butyrate	2.61	6.60	4.20 ± 1.06[2.52-7.69]
	[1-^14^C]-octanoate	1.41	4.77	3.53 ± 0.81[3.53-6.37]
- by carbon dioxide plus water soluble material production	[1-^14^C]-palmitate	5.70	7.39	6.08 ± 1.03[4.28-8.51]
- by tritiated water formation	[9,10-^3^H]-myristic acid	1.03	9.27	7.75 ± 1.73[4.97-11.20]
	[9,10-^3^H]-palmitic acid	1.75	7.41	6.12 ± 1.28[4.00-9.20]

Control values are means ± SD, range control values are given below between brackets.

Biochemical determinations were also performed on patient fibroblast preparations (Table 3). Overall fibroblast fatty acid oxidation rates investigated with either [9, 10-^3^H]-myristic acid or [9, 10-^3^H]-palmitic acid confirmed a fatty acid oxidation defect. Other measurements of mitochondrial oxidation performed on intact fibroblasts indicated that the fatty acid oxidation defect involved medium-chain fatty acid (octanoate) β-oxidation. This location of the defect was confirmed, using either octanoyl-CoA or phenylpropionyl-CoA as a substrate, by the reduction of MCAD activity along with normal VLCAD and SCAD activities. Reduced oxidation of phenylpropionate by intact fibroblasts (10% of control values) further confirmed deficient MCAD activity (data not shown).

**Table 3:** Major biochemical determinations performed on cultured skin fibroblasts from patient.

Metabolic steps	Substrates	Patient	Controls
		(nmol/hr.mg protein)	(nmol/hr.mg protein)
Mitochondrial oxidations assayed			
- by carbon dioxide production	[1,4-^14^C]-succinate**^e^**	3.28	4.08 ± 0.71 (n = 20)
	[1-^14^C]-butyrate**^f^**	7.37	8.83 ± 1.69 (n = 20)
	[1-^14^C]-octanoate**^g^**	1.09	6.75 ± 2.08 (n = 20)
- by carbon dioxide plus water soluble material production	[1-^14^C]-palmitate**^h^**	10.59	10.85 ± 2.60 (n = 20)
- by tritiated water formation	[9,10-^3^H]-myristic acid **^a^**	0.90**^b^**	5.81 ± 2.27 (n = 76)
	[9,10-^3^H]-myristic acid **^a^**,**^c^**	1.03	8.04 ± 1.74 (n = 20)
	[9,10-^3^H]-palmitic acid**^d^**	3.49	9.39 ± 1.70 (n = 20)
Acyl-CoA dehydrogenases (ETF as an electron acceptor)		(nmol/min.mg protein)	(nmol/min.mg protein)
-VLCAD	palmitoyl-CoA**^i^**	0.69	1.3 ± 0.5 (n = 55)
-MCAD	octanoyl-CoA**^j^**	0.48	1.7 ± 0.5 (n = 55)
-SCAD	butyryl-CoA**^k^**	0.71	1.0 ± 0.4 (n = 55)
Enoyl-CoA hydratases	crotonyl-CoA (C4)	307	346 ± 112 (n = 63)
	2-dodecenoyl-CoA (C12)	81	78 ± 25 (n = 59)
	C12/C4 activity ratio	0.26	0.24 ± 0.05 (n = 59)
β-hydroxyacyl-CoA dehydrogenases	acetoacetyl-CoA (C4)	116	99.5 ± 32.1 (n = 105)
	β-keto-palmitoyl-CoA (C16)	74	81.8 ± 22.8 (n = 102)
	C16/C4 activity ratio	0.64	0.86 ± 0.20 (n = 102)
β-ketoacyl-CoA thiolase	β-keto-palmitoyl-CoA (C16)	19.8	20.64 ± 7.79 (n = 47)
	acetoacetyl-CoA (no K^+^)	5.20	6.44 ± 3.46 (n = 60)
	acetoacetyl-CoA (with K^+^)	13.7	13.40 ± 6.22 (n = 60)
	activity ratio AA-CoA with		
	K^+ ^on AA-CoA without K^+^	2.64	2.17 ± 0.59 (n = 60)
Succinyl-CoA Ketoacid Transferase	succinyl-CoA and acetoacetate	10.4	12.11 ± 3.60 (n = 25)
Carnitine palmitoyltransferase			
Type I (outer)	palmitoyl-CoA	0.53	0.58 ± 0.26 (n = 12)
Type II (inner)	palmitoyl-CoA	20.70	15.37 ± 3.13 (n = 8)
Cell membrane carnitine transport	L-carnitine	0.73 10^-3^	0.83 10^-3 ^±0.25 10^-3 ^(n = 8)

Control values are means ± SD

**^a ^** Performed by two laboratories; **^b ^** Conjointly run with fibroblasts from a patient with classical MCADD (homozygous for c.985A>G mutation) and for whom measured activity was 0.4 nmol/hr.mg protein;

**^c^**,**^d ^** Conjointly run with control fibroblasts exhibiting rates of 7.36 and 9.60 nmol/hr.mg protein, respectively; **^e^**,**^f^**,**^g^**,**^h ^** Concomitantly run with control fibroblasts exhibiting rates of 3.29, 8.10, 4.12 and 11.01 nmol/hr.mg protein, respectively; **^i^**,**^j^**,**^k ^** Conjointly run with control fibroblast preparations having activities of 0.70, 1.36 and 0.48 nmol/hr.mg protein, respectively.

Residual MCAD activity studied by the various procedures was between 10 to 28% of control values and overall fatty acid oxidation rates were lowered to similar extents. The activities of other enzymes involved in mitochondrial β-oxidation, including enoyl-CoA hydratases, β-hydroxyacyl-CoA dehydrogenases and β-ketoacyl-CoA thiolases as well as the ketolysis enzyme succinyl-CoA ketoacid transferase, were in normal range values. The proteins involved in cellular utilization of carnitine and its esters, including carnitine palmitoyltransferase types I and II and the plasma membrane carnitine transporter(s) also exhibited normal activities in fibroblast preparations (Table 2).

### Genetic studies

**Figure 1:**
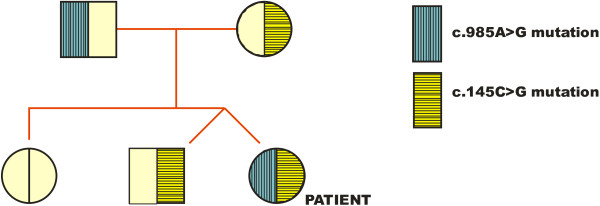
**Pedigree diagram showing haplotypes for the *ACADM *allele mutations in the patient, siblings and parents**.

PCR amplification of all exons of the human *ACADM *gene, including part of the flanking intron sequences, was carried out as previously described [12] using intron-located primers under standard conditions. The PCR products were subjected to bi-directional cycle sequencing using DNA dye terminator sequencing kits (BigDye, Perkin-Elmer). Patient DNA samples were found to be heterozygous for the common c.985A>G mutation in exon 11. Another heterozygous mutation, consisting of C replacement by G at position 145 of the cDNA sequence (c.145C>G) in exon 3 was identified. Sequence analysis of exons 3 and 11 was then performed on DNA samples from parents and siblings. A pedigree tree for the two *ACADM *mutations is shown in Figure 1.

### *De novo* syntheses of acylcarnitines from deuterated palmitate

**Figure 2:**
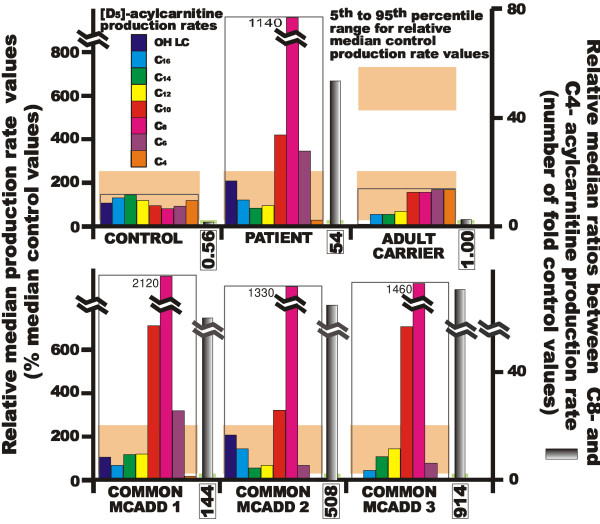
***De novo *individual acylcarnitine synthesis by whole blood samples from the mild MCADD patient, a healthy control and a adult carrier (top panels), and from patients with classical MCADD (bottom panels)**. *De novo *acylcarnitine synthesis rates generated from deuterated palmitate in the presence of added L-carnitine by whole blood samples and derived C8/C4 acylcarnitine production rates have been determined in patient (compound heterozygous for c.985A>G and c.145C>G *ACADM *mutations) and a adult healthy volunteer and is compared to the same exploration performed in a adult carrier for the c.985A>G *ACADM *mutation (see top panels). The series of bottom panels report results of these measurements in individual patients with the classical form of MCADD and with proven homozygocy for the c.985A>G *ACADM *gene mutation. Formations of individual [D5]-acylcarnitines in whole blood samples are relative median rates expressed as percentages of median control values (framed profiles). The ratio between the rates of production of the major C8-acylcarnitine and the C4-acylcarnitine is expressed as relative median units, *i.e*. as the number of fold (and not the number of percents of) median control values. Values of C8 acylcarnitine production rates in disrupted histograms are given at the top of these histograms, and values for C8/C4 acylcarnitine ratios are given below each ratio histogram. The 5^th ^to 95^th ^percentile range for relative median production rate values is given in back position of each framed series of acylcarnitine production rates. The 5^th ^to 95^th ^percentile range for relative median C8/C4 acylcarnitine ratios is represented by the small green rectangle present at the basis of each C8/C4 ratio histogram and was exceeded by all MCADD patient values but neither carrier nor control values.

*De novo *syntheses of acylcarnitines from deuterated palmitate were studied in whole blood samples from the patient and healthy controls. Relative median [D5]-acylcarnitine production rates generated from incubation of whole blood samples with [16-^2^H_3_, 15-^2^H_2_]-[D5]-palmitate along with C8 on C4 acylcarnitine production rate ratio are illustrated in Figure 2 for the patient and a healthy control. Compared with the profile in this healthy control and with median reference values originating from 59 distinct healthy control subjects, the data from our patient with mild MCADD appeared to be pathological. Both the profile of deuterated acylcarnitine produced from stable-labeled palmitate and the ratio between rates of production of C8- and C4-acylcarnitines were abnormal, aligning on profiles in three patients with classical severe MCADD (Figure 2, bottom panels). This datum is clearly distinguishable from profiles in a carrier adult who was heterozygous for the c.985A>G MCAD gene mutation (Figure 2, top panels). The classical MCADD patient 1 was among those reported in previous work [34]; patients 2 and 3 being sister and daughter of the classical MCADD patient 1 and of the adult carrier, respectively.

## Discussion

Our patient undoubtedly suffered from a deficiency of MCAD, presenting with a mild phenotype of the disease as ascertained by relatively high residual activity from lymphocytes and fibroblasts and by a low urinary excretion of hexanoylglycine and suberylgycine during metabolic decompensation. At the time of this urine analysis, acylglycines were determined by GC/MS (with a detection limit of 2 mmol/mol creatine) and could not be discriminated from control values. Better analytical detections of urinary acylglycines are obtained by GC/MS-MS or GC-MS stable-isotope dilution analysis (SID) of methyl esters, with detection limits less than 0.7-1 mmol/mol creatinine, allowing for upper limit of controls a clear cut-off value close to 1.1 mmol/mol [25,41].

Biochemically, our patient resembled the four mild MCADD patients described by Zschocke et al. [25] who had a residual MCAD activity (10-20%) of the same order of magnitude as in our patient. In fact, these four cases and our patient all show intermediate (between controls and severe MCADD patients) values for both enzyme activities and acylcarnitine levels and ratios. The residual enzyme activity observed in classical MCADD, caused by homozygosity for c.985A>G, is usually below 1%. Two of the four patients previously described by Zschocke et al. [25] were homozygous for *ACADM *gene mutations (c.734C>T or c.799G>A [exon 9]) distinct from the c.985A>G mutation; the other two patients were compound heterozygous for the c.985A>G mutation in one allele and the c.199T>C (exon 3) mutation in the other *ACADM *allele. Genotypically, our patient resembled the latter two patients, with the c.985A>G mutation (exon 11) in one allele and a mutation in exon 3 (namely, c.145C>G mutation) in the other allele. This mutation has never been observed in controls. To our knowledge, this is the first report for the c.145 C>G mutation in the *ACADM *gene.

As a general rule, amino acid changes introduced in a mature protein affect its conformation and this may impact its final stability and function/activity. This has been well documented for the MCAD mutant protein (K304E) resulting from the c.985A>G mutation. The MCAD protein requires assembly of the monomers in a tetramer, which represents the active functional form of MCAD. In the case of monomers resulting from the wild-type *ACADM *gene, the MCAD tetramer is stable. In the case of K304E monomers, the tetramer dissociates abnormally rapidly [26], giving rise to a reduced steady-state concentration of the tetrameric form. Another contributing factor is impairment of intramitochondrial processes including altered monomer folding or management [27]. Several studies indicate that protein misfolding is the key event in the loss of MCAD activity from many protein variants, and that overexpressing chaperonins may rescue monomers from aggregation and enhance residual MCAD activity (see [3] and references therein). As a result of protein misfolding, accelerated temperature-driven unfolding may affect MCAD protein variants. This abnormal MCAD loss of function and thermosensitivity first documented for the K304E variant, has been recently extended to a number of MCAD variants [18,42], questioning the view that some of the missense mutations detected by newborn screening are less life-threatening than those clinically proven to be disease causing. In this respect, the relatively high residual activity found in our mild MCADD patient has not prevented the occurrence of a severe metabolic decompensation which might have been fatal in the absence of adequate medical management. A patient with the R28C mutant protein who had a mutation (c.157C>T), also less severe than the c.985A>G mutation, died unexpectedly in early childhood [43].

Our mild MCADD patient combines MCAD K304E and Q24E variant monomers. Conformational characteristics of the MCAD K304E variant have been repeatedly described in the literature. The amino acid change is located in the MCAD C terminal α domain which is involved in helix-helix interactions taking place in tetramer assembly (see [18] and references therein). As a result, tetramer assembly is compromised: unemployed monomers aggregate and are subjected to degradation. Experimental prevention of monomer aggregation by overexpressed chaperonins has been shown to result in increased tetramer assembly along with higher residual activity (see [3] and references therein).

The Q24E variant has not been previously described. The amino acid change here occurs in the MCAD N-terminal α domain. Some of the protein variants with mutations in this protein region may present with normal or close to normal enzyme activity [3,18]. Mutations of this portion of the MCAD protein are also shown to induce moderate alterations in tetramer assembly and to disrupt thermal stability. This is the case for the Y42 H mutant protein for which temperature-driven loss of activity is greater than normal though lower than that of common K304E mutant protein [42]. *De novo *synthesis of acylcarnitines monitored on whole blood samples from our patient was consistent with MCADD. The major acylcarnitine produced from deuterated palmitate was octanoylcarnitine, other medium-chain (hexanoyl- and decanoyl-) carnitine esters being also generated in excess. The C8 to C4 acylcarnitine production rate ratio was 54-fold that of controls. As a whole, profile and ratio of acyl-carnitine production rates in our mild MCADD patient resemble those found in patients with classical MCADD, indicating clearly functional repercussion of the enzyme defect on the disposal of acyl-CoA. The carnitine added to incubation media appeared to mobilize acyl-CoAs unmetabolised by β-oxidation and, by comparing acylcarnitines production rates to control values, revealed the level(s) at which β-oxidation was defective. The acylcarnitine production rates and increased C8/C4 acylcarnitine *de novo *synthesis ratio of our mild MCADD patient are clearly different from those of the adult carrier of the c.985A>G mutation (figure 2).

The last but not least issue concerns the carnitine status of our patient at the time of metabolic decompensation. The patient was then not receiving L-carnitine therapy and her systemic carnitine levels were below normal (Table 1). This chronic hypocarnitinaemia was concomitant with a low urinary excretion of hexanoylglycine and suberylglycine. Interestingly, no elevation of urinary suberylglycine was reported in the urine from one patient with hypocarnitinaemia in the description of four MCADD asymptomatic siblings (compound heterozygotes for the common c.985A>G and the c.842G>C ACADM gene mutations) provided by Albers and coworkers [44]. Zschocke and coworkers on the other hand discussed elevated acylcarnitine and undetectable 5-hydroxyhexanoic acid and suberylglycine in their four mild MCADD patients [25]. Patients with genotypes representing mild MCADD (high residual enzyme activity and low urinary levels of glycine conjugates) should get the same advice as patients with classical MCADD regarding risks associated with catabolic states and carnitine deficiency.

## Competing interests

The authors declare that they have no competing interests.

## Authors' contributions

AFD and MF contributed equally to this work. AFD, MF, AMP, GB and JV designed and performed measurements of *de novo *acylcarnitine syntheses by whole blood samples. BSA and NG contributed to genetic studies. MB, CVS and RJAW performed the biochemical and enzyme measurements. SNG, DD and KMM contributed to the clinical follow-up of studied patients. DR determined organic acids in body fluids and DSM performed acylcarnitine analysis on dried blood spots. Each author read the text and JV coordinated the writing of the manuscript. All authors read and approved the final manuscript.

## Consent

Written informed consent was obtained by mother of the patient for publication of this case report.
